# Interaction of nanoparticles with motile gyrotactic microorganisms in a Darcy-Forchheimer magnetohydrodynamic flow- A numerical study

**DOI:** 10.1016/j.heliyon.2023.e17840

**Published:** 2023-07-05

**Authors:** Faiz Muhammad, Tahar Tayebi, Kashif Ali, Emad Hasani Malekshah, Sohail Ahmad

**Affiliations:** a Department of Mathematics, Faculty of Sciences, The University of Faisalabad, Faisalabad, Pakistan; b Department of Mechanical Engineering, Faculty of Sciences and Technology, University Mohamed EL Bachir EL Ibrahimi of Bordj Bou Arreridj, El-Anasser, 34030, Algeria; c Department of Basic Sciences and Humanities, Muhammad Nawaz Sharif University of Engineering and Technology, Multan, Pakistan; d Department of Power Engineering and Turbomachinery, Silesian University of Technology, 44-100 Gliwice, Poland; e Centre for Advanced Studies in Pure and Applied Mathematics (CASPAM), Bahauddin Zakariya University, Multan, 60800, Pakistan

**Keywords:** Gyrotactic microorganisms, Nanoparticles, Darcy-Forchheimer flow, Heat generation, Chemical reaction, Successive over relaxation method

## Abstract

The present work aims to interpret the mass and heat transferal flow through Darcy Forchheimer porous medium involving, simultaneously, microorganisms and nanoparticles. The involvement of gyrotactic microorganisms in the flow of nanoparticles reinforces the thermal characteristics of several energy systems. The amalgamation of microorganisms (microbes) in the nanofluids not only enhances the thermal properties of the fluid but it also causes the stability in the flow. Some other prominent effects such as chemical reaction and heat generation have also been taken into account. The reduced form of the governing model equations is further simplified in order to obtain the algebraic system of equations. Afterward, the approximate solution is obtained by developing an algorithm in the MATLAB software. To check the validity and efficiency of code, we have correlated our numerical outcomes with the previously accomplished ones. The outcomes are explained via the tabular and graphical representations. The flow of nanofluids will be more stable if it involves the motile microorganisms. Another example of the utilization of microorganisms is the microbial-enhanced oil recovery. In order to maintain the variation in the oil bearing layers, the microorganisms along with other nutrients can be incorporated. A significant enhancement is noticed in temperature in case of an increase in the values of heat generation and thermophoresis parameter.

## Nomenclature

$k^{*}$: darcy permeability
$D_{B}$: Brownian motion diffusivity
$Nu_{x}$: Nusselt number
$N_{\infty }$: ambient microorganisms concentration
N: microorganisms concentration
$D_{n}$: diffusivity of microorganisms
$C_{\infty }$: ambient concentration
$u_{e}$: ambient velocity
τ: heat capacity of nanofluid
η: similarity variable
$\tilde{\alpha }$: thermal conductivity
$Sh_{x}$: Sherwood number
θ: dimensionless temperature
C: fluid concentration
$C_{b}$: drag force coefficient
$W_{c}$: cell swimming speed
ρ: fluid density
m: positive constant for nonlinear stretching sheet
$C_{fx}$: skin friction
ψ: stream function
μ: dynamic viscosity
a,c: Constants
$T_{\infty }$: ambient temperature
$D_{T}$: thermophoretic coefficient
$Nn_{x}$: density of motile microorganisms
T: fluid temperature
$K_{c}$: rate constant of chemical reaction
$u_{w}$: stretching velocity
υ: kinematics viscosity

## 1 Introduction

The motile microorganisms and nanofluids have extensively been studied by the research community as they, combined, have potential uses in modern bio-technology. The combination of small sized solid particles and the base liquids like benzene, water, ethylene glycol gives rise to the nanofuids. These fluids work as heat transferring agents in many energy systems. Initially, it was claimed by Choi [1] that the mixture of solid nano particles of metals or metallic oxides would sufficiently increase the thermal conductivity of the fluid. This idea provided the base of further exploration of nanofluids. It was proposed that inserting the nanomaterial into the base fluid would cause an augmentation in the thermal characteristics of the specified (base) fluids. The same idea was adopted by several researchers who further interpreted the novel type of nanomaterial [[2], [3], [4], [5], [6], [7], [8], [9]]. Other than the cooling and heating processes in many engineering systems, the nanofluids have been employed in nuclear cooling, refrigeration, engine cooling, energy reservoirs, tumor and cancer therapy, Car AC, high power lasers, pharmacology, microwaves tubes and so on. The different concentrations of nanoparticles show improvement in the thermal characteristics of the fluids and, the suspension of nanoparticles into the host fluid gives rise to the nanofluid. A lot of work is available which describes the nanofluids’ behavior in augmentation of the thermal characteristics of industrial and engineering mechanisms. The improvement in heat transfer by nanofluids is required essentially in many industrial applications like biomedicines, nuclear reactors, electronics and transportation.

Several practical applications include thermophysical nanofluid features created by a suspension of nano-particles with escalating behavior. The structural features of nanoparticles in the base fluid are mainly responsible for the efficient thermal conductivity created. Xian et al. [10] selected ethylene glycol and water from distillation as a base liquid for combined nanofluids containing Graphene nanoplatelets (GNPs) and TiO_2_. Urmi et al. [11] investigated the thermophysical features of 40% ethylene glycol-based TiO_2_–Al_2_O_3_ hybrid nanofluid. Thermal features of Cu-based nanofluid contenting surfactant molecules are investigated by Abu-Hamdeh et al. [12]. It was found that the thermal conductivity of the nanofluid containing the surfactant molecules is higher than the Cu-based nanofluid. Singh et al. [13] evaluated the thermal conductivity and specific heat capacity of oil TH-66/MWCNT-based nanofluids for different weight fractions of 0.05–0.5 wt%, pressure up to 70 bar, and a temperature range of 25–300 °C. Mosavi et al. [14] investigated the thermal properties of Cu–Ar nanoliquids with atomic barriers between simulated platinum plates. According to findings, increasing the atomic barrier radius enhances thermal properties. Ali et al. [15] carried out an excellent investigation on the thermophysical characteristics of several nanofluids in interesting situations.

Kuznetsov [16] was the first who initiated the abstract work of nanofluid flow encompassing motile gyrotactic microorganisms. He observed the self-propelled motion of living organisms in the nanofluid flow. It was noticed that the microorganisms were moving on the upper surface of boundary layer flow. It may be because of the reason that microorganisms are denser than water and this phenomenon force the microbes to move in the upward direction. This phenomenon is not the same in all conditions as the participation of various influences like thermal radiation and viscous dissipation will change the movement of microbes. The self-swimming of microbes can also be interrupted by some other factors e.g., viscous and gravitational torques. The analysis of both hybrid and pure nanofluids in the presence of microorganisms was presented by Ahmad et al. [17]. Raza et al. [18] and Faizan et al. [19] investigated the Sutterby nanofluid flows involving gyrotactic microorganisms over a stretchable cylinder and Riga sheet respectively. Bhatti et al. [20], Abdelsalam et al. [21], and Alsharif et al. [22] interrogated the thermal behavior of nanofluids under several effects. Shahid et al. [23] applied the “Successive Taylor Series Linearization Method (STSLM)” to calculate of the boundary layer flow of microorganisms and nanofluids. It was elaborated that how much boundary layer flow was exaggerated by the magnetic interaction parameter.

Ramzan et al. [24] premeditated the influences of nonlinear thermal radiation and velocity slips on the bio-convective nanofluid flow. In this research, the impacts of two bioconvection parameters named Peclet number and Lewis number were found to be prominent in the density distribution of microbes and fluid's velocity. The magnetohydrodynamic effect was taken into account by Chakraborty et al. [25] to examine the role of tiny-sized solid nanoparticles and gyrotactic microorganisms in the nanofluid flow. The magnetic field interaction parameter caused a reduction in the mass flux rate of microbes. Aziz et al. [26] and Iqbal et al. [27] planned the bioconvective stagnation point flow having heat and transferal characteristics. Tausif et al. [28] studied some applications of the bioconvective flow in polymer industry. The increasing values of slip parameters interrupted the thermal and flow characteristics of the boundary layer flow.

The major aim to study this paper is to examine the flow and thermal features of Darcy Forchheimer flow that involves both nanoparticles and gyrotactic microorganisms in coexistence of some other effects such as chemical reaction, heat generation and magnetohydrodynamic. We describe some distinguish features of our work as:

➢ It is revealed from the existing literature review that less attention is paid towards the computational analysis of gyrotactic microorganisms and nanofluid subject to nonlinear Darcy porous medium.
➢ The current investigation has the purpose of filling this gap & inspection of nanofluid flow involving gyrotactic microorganisms.
➢ We have developed a persuasive numerical algorithm to find the approximate solutions of the problem.
➢ The governing system of transmuted equations is numerically solved via this method which gives faster convergence.
➢ The consequences of our study are elaborated through tables and graphs.

## 2 Mathematical description

**Fig. 1:**
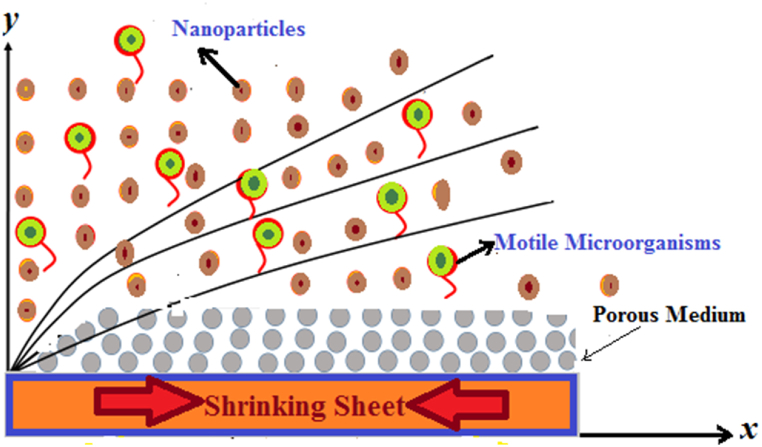
Physical problem.

Let us assume the nanofluid flow subject to Darcy Forchheimer porous medium over a nonlinear extending surface. Nanoparticles and gyrotactic microorganisms are present in the flow. The combined effect of chemical reaction as well as heat generation is also taken into account. Both $C_{w}$ and $N_{w}$ represent, respectively, the concentrations of fluid and microorganisms. Whereas the fluid's temperature on the surface of the sheet is expressed by $T_{w}$. The same terms away from the surface can be represented as $\left(T_{\infty },C_{\infty },N_{\infty }\right)$. The microbes' path way for swimming is not influenced by the presence of nano-sized solid particles. But the movement of microorganisms may be interrupted in case the concentration in volume of nanoparticles is greater than1%. The amalgamation of the gyrotactic microorganisms and nanoparticles will give rise to the stability of the flow. The boundary layers across the flow zone are depicted in Fig. 1.

The equations governing the problem under the above assumptions (in case of Darcy Forchheimer medium, chemical reaction and heat generation) have the following form [[24], [29], [30]]:

(1) $$\frac{\partial v}{\partial y}+\frac{\partial u}{\partial x}=0$$

(2) $$u\frac{\partial u}{\partial x}+v\frac{\partial u}{\partial y}=\upsilon \frac{\partial^{2}u}{\partial y^{2}}+u_{e}\frac{\partial u_{e}}{\partial x}+\frac{\mu }{\rho k^{*}}\left(u_{e}-u\right)+\frac{\sigma B_{0}^{2}}{\rho }\left(u_{e}-u\right)-\frac{C_{b}}{\sqrt{k^{*}}}u^{2}$$

(3) $$u\frac{\partial T}{\partial x}+v\frac{\partial T}{\partial y}=\frac{\tau D_{T}}{T_{\infty }}{\left(\frac{\partial T}{\partial y}\right)}^{2}+\tilde{\alpha }\frac{\partial^{2}T}{\partial y^{2}}+\frac{Q_{0}}{\rho c_{p}}\left(T-T_{\infty }\right)+\tau D_{B}\frac{\partial C}{\partial y}\frac{\partial T}{\partial y}$$

(4) $$v\frac{\partial C}{\partial y}+u\frac{\partial C}{\partial x}=D_{B}\frac{\partial^{2}C}{\partial y^{2}}+\frac{D_{T}}{T_{\infty }}\frac{\partial^{2}T}{\partial y^{2}}-K_{c}\left(C-C_{\infty }\right)$$

(5) $$v\frac{\partial N}{\partial y}+u\frac{\partial N}{\partial x}+\frac{1}{C_{w}-C_{\infty }}\frac{\partial }{\partial y}\left[N\left(\frac{\partial C}{\partial y}\right)\right]bW_{c}=D_{n}\frac{\partial^{2}N}{\partial y^{2}}$$

The coordinates of the axes are expressed by x and y which are taken respectively along and across the surface with velocity components u and v. The Proposed boundary conditions (BCs) at y=0 (on the surface) and y→∞ (away from the surface) are composed as:

(6) $$y=0:u=cx^{m},C=C_{w},N=N_{w},v=v_{w}\left(x\right),T=T_{w}$$

(7) $$y\to \infty :u=ax^{m},C=C_{\infty },N=N_{\infty },T=T_{\infty }$$

Furthermore, the terms in the above equations are mentioned as: $k^{*}$ is the Darcy permeability, $C_{b}$ is the drag force coefficient, $D_{B}$ is the Brownian diffusion coefficient, T is the temperature, $D_{n}$ is diffusivity of microorganisms, $c_{p}$ is the specific heat constant, $\tilde{\alpha }$ is the thermal conductivity, υ is kinematic viscosity, N is the microorganisms concentration, ρ is the density, τ is the nanofluid heat capacity ratio, C is the concentration, $bW_{c}$ is the cell swimming speed, $D_{T}$ is the thermophoretic coefficient, $K_{c}$ is the rate constant of chemical reaction.

In order to transform equations (1), (2), (3), (4), (5) into ODEs, we adopt the following dimensionless coordinates, in equation (8) [31],:

(8) $$\psi =\sqrt{ax^{m+1}\upsilon }f\left(\eta \right),\theta \left(\eta \right)=\frac{T-T_{\infty }}{T_{w}-T_{\infty }},\xi =\sqrt{\frac{a}{\upsilon }x^{m-1}}y,\varphi \left(\eta \right)=\frac{C-C_{\infty }}{C_{w}-C_{\infty }},G\left(\eta \right)=\frac{N-N_{\infty }}{N_{w}-N_{\infty }}$$

We achieve the following dimensionless system of equations (9), (10), (11), (12) by entreating above variables:

(9) $$f^{\prime \prime \prime }+\left(M+P_{0}\right)\left(1-f^{\prime }\right)=\left(m+D_{f}\right){f^{\prime }}^{2}-m-\frac{1+m}{2}ff^{\prime \prime }$$

(10) $$\frac{1}{\mathrm{Pr}}\theta^{\prime \prime }+N_{b}\varphi^{\prime }\theta^{\prime }+\frac{1+m}{2}f\theta^{\prime }+H_{0}\theta +N_{t}{\theta^{\prime }}^{2}=0$$

(11) $$\varphi^{\prime \prime }-LeR_{c}\varphi =-\frac{N_{t}}{N_{b}}\theta^{\prime \prime }-\frac{1+m}{2}Lef\varphi^{\prime }$$

(12) $$G^{\prime \prime }+\frac{1+m}{2}fScG^{\prime }=Pe\left[G^{\prime }\varphi^{\prime }+\varphi^{\prime \prime }\left(\Omega +G\right)\right]$$

The boundary conditions (6) and (7), in the light of similarity variables, take the following form in equation (13):

(13) $$\begin{array}{l} \xi =0:S=f=1,\varphi =1,f^{\prime }=\alpha ,\theta =1\\ \xi \to \infty :\varphi \to 0,\theta \to 0,f^{\prime }\to 1,G\to 0 \end{array}$$

Where α represents the shrinking/stretching of the surface depending on the conditions α<0 (shrinking case) or α>0 (stretching case) and S relates to the suction/injection parameter. The system of equations (9), (10), (11), (12) involves the dimensionless parameters defined in equation (14) as:

(14) $$\begin{array}{l} \varepsilon_{0}=\frac{\nu x}{k^{*}u_{e}},N_{b}=\frac{\tau D_{B}\left(C_{w}-C_{\infty }\right)}{\nu },\Omega =\frac{N_{\infty }}{N_{w}-N_{\infty }},Sc=\frac{\nu }{D_{n}},H_{0}=\frac{Q_{0}x}{\rho c_{p}u_{e}}\;N_{t}=\frac{\tau D_{T}\left(T_{w}-T_{\infty }\right)}{\nu T_{\infty }}\\ Le=\frac{\nu }{D_{B}},\mathrm{Pr}=\frac{\nu }{\alpha },Pe=\frac{bW_{c}}{D_{n}},M_{ag}=\frac{\sigma B_{0}^{2}x}{\rho u_{e}},\lambda_{CR}=\frac{2K_{c}x}{u_{e}},D_{F}=\frac{C_{b}x}{\sqrt{k^{*}}}\text{.} \end{array}\}$$

We introduce the following quantities of engineering interests represented in equation (15) which denote the Sherwood number, motile microbes’ density number, surface drag, and Nusselt number:

(15) $$Nu_{x}{Re}_{x}^{-\frac{1}{2}}=-\theta^{\prime }\left(0\right),{Re}_{x}^{-\frac{1}{2}}Sh_{x}=-\varphi^{\prime }\left(0\right),{Re}_{x}^{\frac{1}{2}}C_{fx}=f^{\prime \prime }\left(0\right),{Re}_{x}^{-\frac{1}{2}}Nn_{x}=-G^{\prime }\left(0\right)\text{.}$$

Where Reynolds number is described by ${Re}_{x}=U_{e}x/\nu$.

## 3 Numerical approach

It may cause trouble while determining the analytical solution of equations (9), (10), (11), (12) as these are coupled and nonlinear equations. That is why approximate solution is the best choice to tackle this problem smoothly. If someone adopts a classical approach (usual numerical schemes) to solve the system of coupled differential equations then it may also create trouble in the case that the equations involve complex Eigen values or the boundary conditions are positioned at infinity. However, we are committed to employing the Successive Over Relaxation (SOR) procedure to determine the approximate resolution to the current problem. This technique does not face hurdles and solves directly the dimensionless system of equations without providing any initial guesses (see details in Ahmad et al. [[32], [33], [34]]). We reduce equations (9), (10), (11), (12) in the following form (equations (16), (17), (18), (19)) and then initiate the iterative process:

(16) $$s_{i}=\frac{1}{P_{1}}\left(Q_{1}q_{i+1}+R_{1}q_{i-1}+S_{1}\right)$$

(17) $$\theta_{i}=\frac{1}{P_{2}}\left(Q_{2}\theta_{i+1}+R_{2}\theta_{i-1}\right)$$

(18) $$\varphi_{i}=\frac{1}{P_{3}}\left(Q_{3}\varphi_{i+1}+R_{3}\varphi_{i-1}+S_{3}\right)$$

(19) $$G_{i}=\frac{1}{P_{4}}\left(Q_{4}g_{i+1}+R_{4}g_{i-1}-S_{4}\right)$$

The structural sketch of our method is given below:

**Figure:**
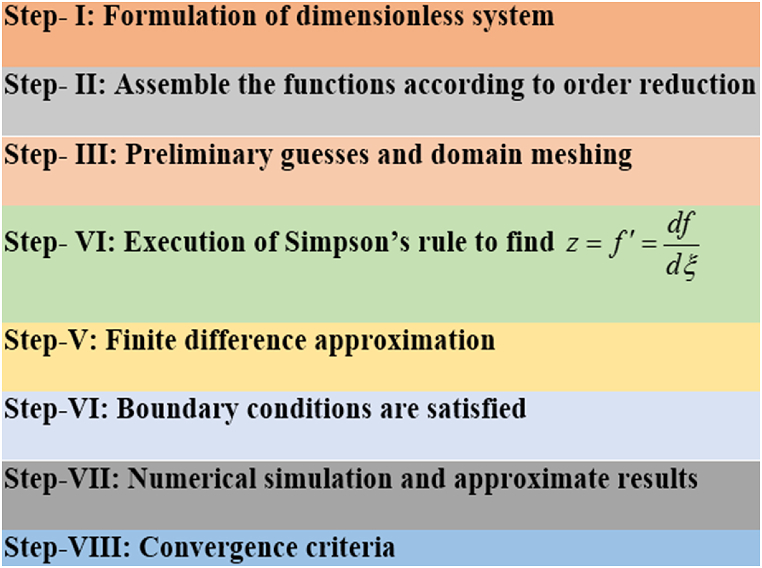

A numerical comparison is used to validate the solution code in limiting cases. The results exhibit better correlation with the earlier ones, as illustrated in Table 1. We gain an assurance of the accuracy of our iterative results from this assessment. Table 1 also deduces that the mass transfer rate of motile microorganisms increases as the suction parameter increases. The uncertainty analysis in the numerical solutions is portrayed in Table 2.

**Table 1:** Comparison and code validation when $D_{f}=H_{0}=\varepsilon_{0}=0$.

S	$Nn_{x}$		
	Ahmad et al. [35]	Zaimi et al. [36]	Present Results
2.5	5.48217	5.48217	5.4748
3.0	6.4984	6.4984	6.4862
3.5	7.54285	7.54285	7.5237
4.0	8.60180	8.60180	8.5736
4.5	9.66858	9.66858	9.6289

**Table 2:** The uncertainty analysis in the numerical calculations.

f(η)					
η	Numerical Sol. for $n=n_{1}$	Numerical Sol. for $n=2n_{1}-1$	Uncertainty from $n=n_{1}\;\text{to}\;n=2n_{1}-1$	Numerical Sol. for $n=4n_{1}-3$	Uncertainty from $n=2n_{1}-1\;\text{to}\;n=4n_{1}-3$
0	0.2000000000	0.20000000000	0	0.20000000000	0
0.5	0.1310789763	0.13078266647	0.00029630990	0.13050110079	0.000281565680456
1.0	0.1121825728	0.111209986153	0.0009725867017	0.110302468344	0.000907517809044
1.5	0.1287474710	0.126742709612	0.0020047613915	0.124897830652	0.001844878959986
2.0	0.1702377153	0.166775453193	0.0034622621373	0.163616645285	0.003158807907695
2.5	0.2291994713	0.223847494749	0.0053519765715	0.218987692106	0.004859802642219
3.0	0.3004810475	0.292896110690	0.0075849368608	0.286023528876	0.006872581814119

## 4 Results and discussion

The numerical outcomes will be interpreted in this section via the graphical and tabular representations. The flow and thermal characteristics, for the prime parameters, are elaborated. We have chosen distinct parametric values rather than assigning the specific values to the parameters. In this way, the desired consequences might be attained in the factual applications of the work. The parametric values that have been taken in the simulation or iterative process are:

$$M_{ag}=3;\;\varepsilon_{o}=4;\;m=2;\;D_{F}=0.2;\;\mathrm{Pr}=6.2;\;Nb=0.3;\;Nt=0.25;\;H=0.2;\;Le=2;\;\lambda_{CR}=1;\;Sc=1.5$$

**Fig. 2:**
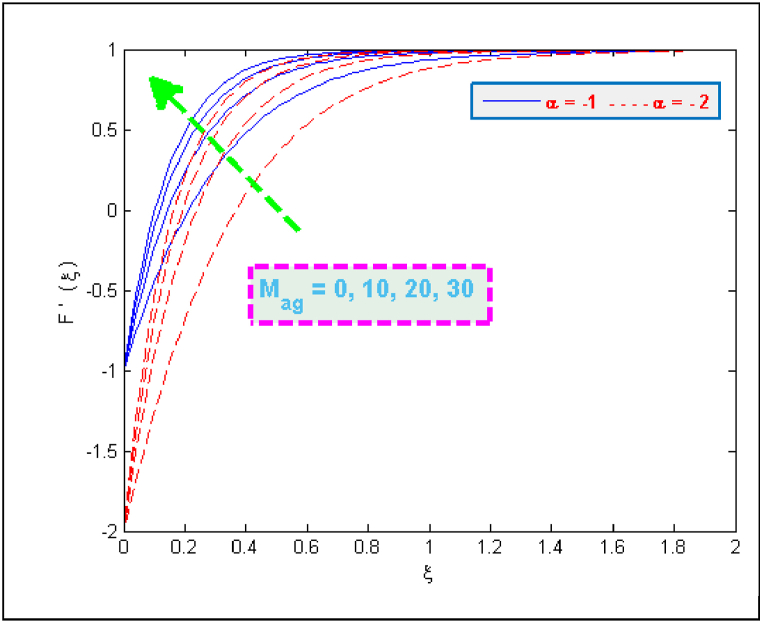
Variation in velocity for several values of $M_{ag}$ and α.

**Fig. 3:**
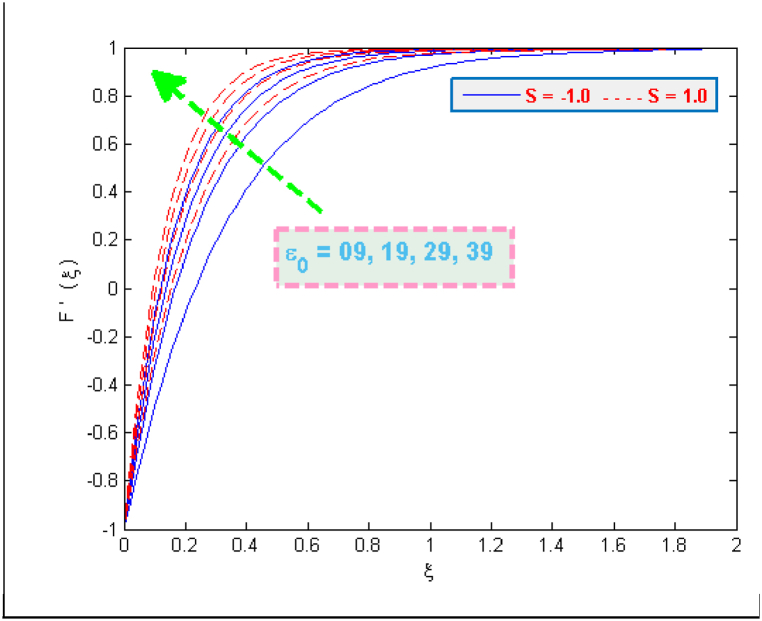
Variation in velocity for several values of $\varepsilon_{0}$ and S.

**Fig. 4:**
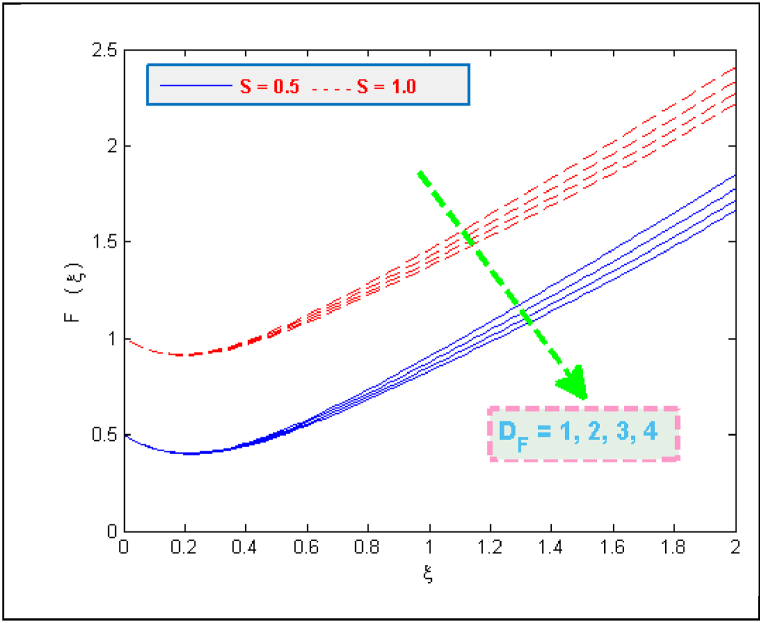
Variation in velocity for several values of $D_{F}$ and S.

**Fig. 5:**
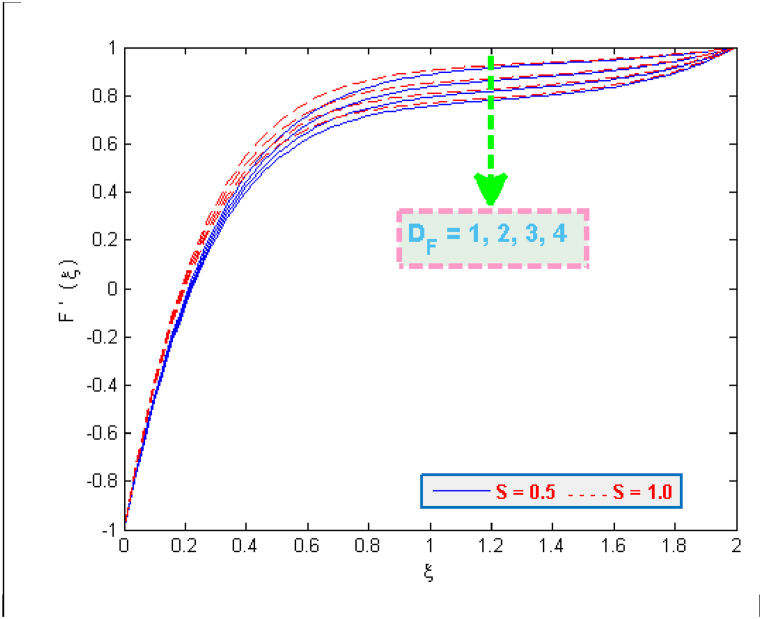
Variation in velocity for several values of $D_{F}$ and S.

Pe=0.4;Ω=0.2;α=−1;S=1;

The results of Fig. 2 expose the fact that shrinking of surface together with magnetic interaction parameter cause an acceleration in the flow velocity. A usual effect of porosity parameter is to deteriorate the fluid's velocity but the present case involves the other side of this fact (see Fig. 3). The presence of microorganisms definitely affects the flow. One of the reasons for this fact is the tenement of microbes in base fluid. The microbes, on the other side, are denser than water. It may also be the reason of increasing velocity. Taking high porosity will allow the particles to pass through porous medium easily. Fig. 4, Fig. 5 portray that how much velocities (F(ξ)& $F^{\prime }\left(\xi \right)$) are affected by the parameter $D_{F}$. Both velocities are reduced due to the reason that when fluid passes through small holes of Darcy Forchheimer medium then it loses its speed. That is why both normal F(ξ) and streamwise velocity $F^{\prime }\left(\xi \right)$ are decelerated by the Darcy Forchheimer parameter $D_{F}$.

The Darcy Forchheimer parameter reduces motile microbes’ density number, Sherwood, Nusselt and skin friction. Whereas the porosity and magnetic parameters act opposite to that of Forchheimer parameter, that is, these parameters have an increasing effect on the physical quantities (See Table 3). These three parameters have substantial effect only on skin friction rather than thermal and mass transfer rates. It may happened due to the fact that the Forchheimer, porosity and magnetic terms are involved only in the momentum equations.

**Table 3:** Change in skin friction, Nusselt, Sherwood and microorganisms’ density numbers with the change in Dracy Forchheimer parameter, porosity parameter and magnetic parameter.

$D_{F}$	$\varepsilon_{0}$	$M_{ag}$	$C_{fx}$	$Nu_{x}$	$Sh_{x}$	$Nn_{x}$
1			7.2916	−3.1604	−1.4423	−2.9633
2			7.1531	−3.1610	−1.4245	−2.9439
3			7.0291	−3.1613	−1.4095	−2.9276
4			6.9154	−3.1616	−1.3966	−2.9134
	09		8.9060	−3.1918	−1.5038	−3.0508
	19		11.2256	−3.2351	−1.5544	−3.1334
	29		13.0896	−3.2656	−1.5837	−3.1845
	39		14.6914	−3.2892	−1.6033	−3.2205
		0	6.3135	−3.1332	−1.4181	−2.9189
		10	9.4242	−3.2021	−1.5168	−3.0715
		20	11.6260	−3.2419	−1.5614	−3.1454
		30	13.4274	−3.2707	−1.5882	−3.1926

**Fig. 6:**
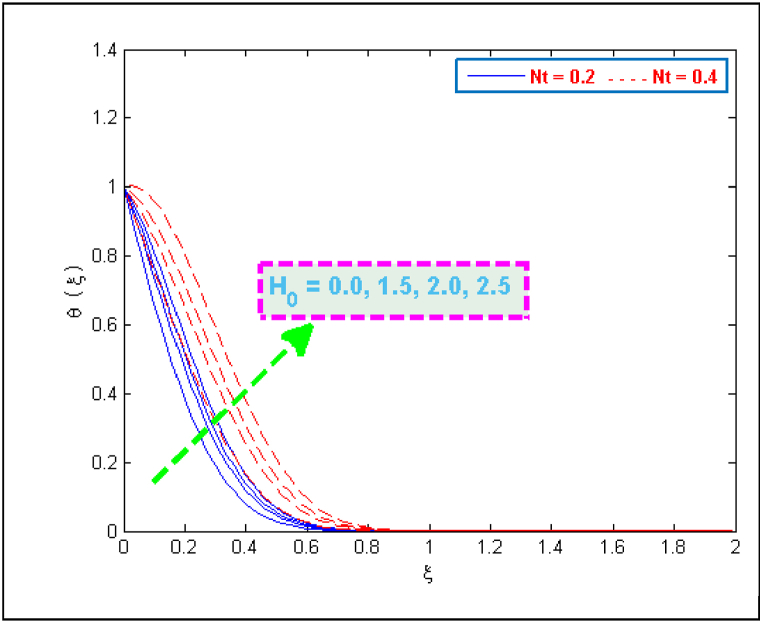
Variation in temperature for several values of $H_{0}$ and $N_{t}$.

**Fig. 7:**
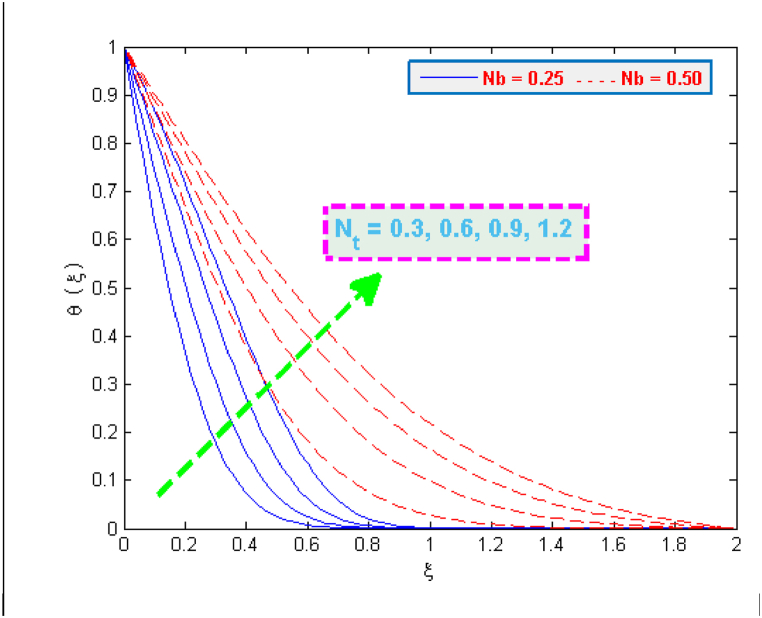
Variation in temperature for several values of $N_{t}$ and Nb.

**Fig. 8:**
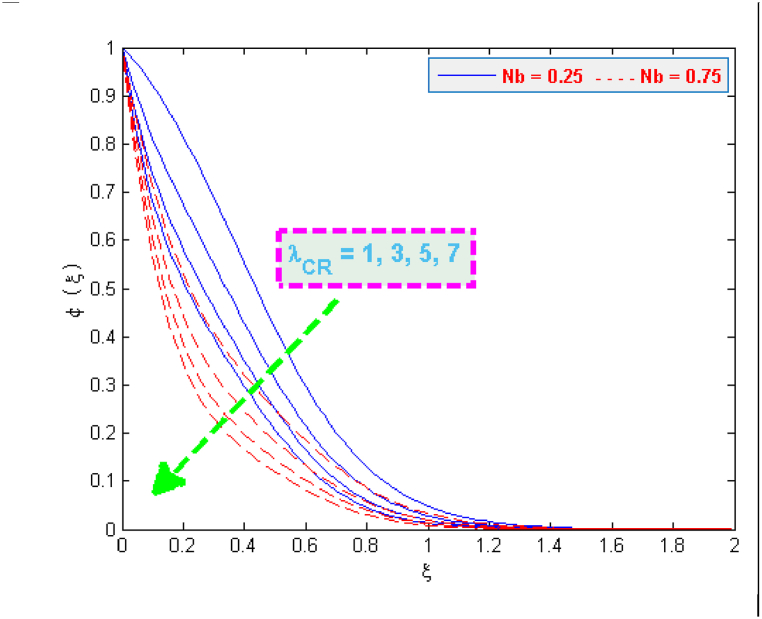
Variation in concentration for several values of $\lambda_{CR}$ and Nb.

**Fig. 9:**
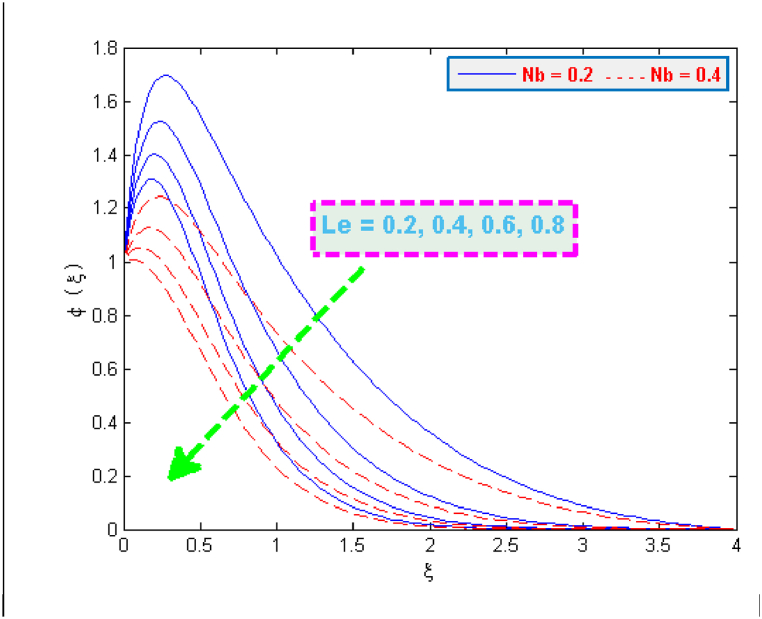
Variation in concentration for several values of Le and Nb.

A significant enhancement is noticed in temperature in case of an increase in the values of heat generation and thermophoresis parameter, as depicted in Fig. 6, Fig. 7. The transfer of heat to the fluid is quicker due to heat generation and consequently this phenomenon raises the temperature. The effect of thermophoretic force is greater near the surface of sheet as compared to its effect away from the surface. However, more heated fluid travels from the surface to away from the surface which is the factor of an increase in temperature. The variation in the concentration profiles for the parameters like chemical reaction $\lambda_{CR}$, Brownian motion Nb and Lewis Le can be observed from Fig. 8, Fig. 9. The concentration profiles seem to be declining with the effect of these three parameters. This fact is attributed by the viscous diffusion rate which when enhances then it reduces the concentration. The mass diffusion of microorganisms also causes the reduction in the volume concentration of nanoparticles which further reduces the concentration of fluid.

**Fig. 10:**
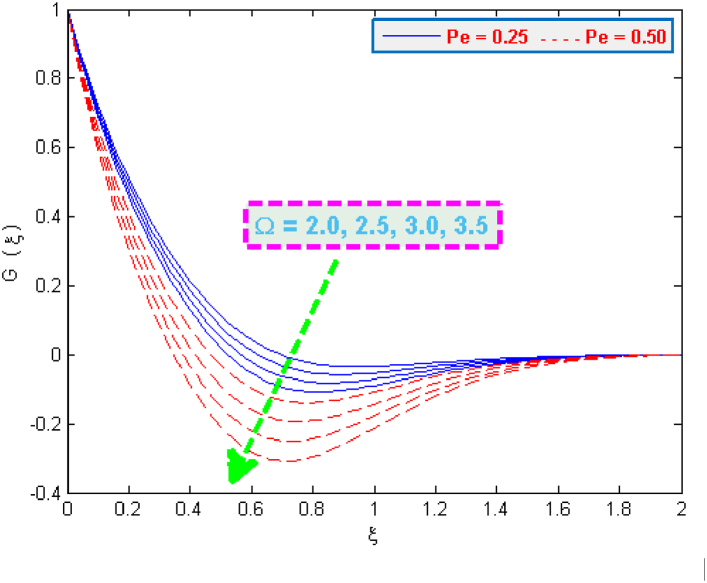
Microorganisms density profile for several values of Ω and Pe.

**Fig. 11:**
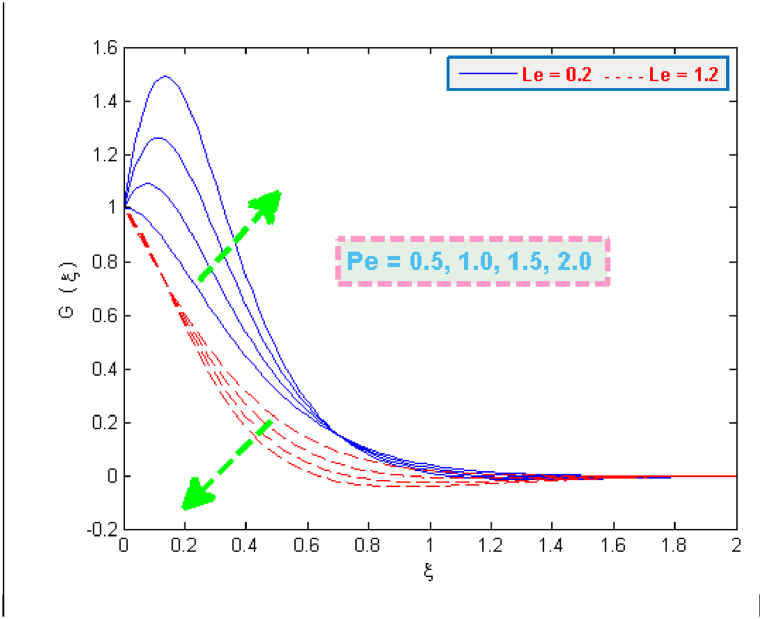
Microorganisms density profile for several values of Pe and Le.

The change in density distribution G(ξ) of microorganisms is portrayed in Fig. 10, Fig. 11. It is evident from these figures that the microbes’ density G(ξ) depresses with an increment in the values of bioconvection parameters e.g. Lewis Le, motile microbes Ω and Peclet Pe. The self-swimming speed of organisms is observed higher on sheet surface because of the enhanced Peclet numbers. But this phenomenon tend to reduce the distribution G(ξ).

It was observed from Table 4 that heat transport rate is reduced by the thermophoretic and heat generation parameter. More heat is produced due to heat generation phenomenon in the fluid. Due to which temperature of fluid magnifies but rate of heat transport reduces. Table 5 depicts the change in mass transfer rate for distinct parameters. The values of Sherwood numbers seem to be elevating with the extending values of the chemical reaction and Brownian motion, as appeared in Table 5. On the other side, the Lewis number is responsible for the decrement in the Sherwood number. Nanofluid's diffusivity is got diminished with the effect of Lewis number which is the main reason of reduction in the Sherwood number.

**Table 4:** Change in Nusselt number with the change in heat generation and thermophoresis parameter.

$H_{0}$	$N_{t}$	$Nu_{x}$
0.0		−3.3429
1.5		−1.6810
2.0		−0.8759
2.5		0.2337
	0.3	−2.9251
	0.6	−1.8323
	0.9	−1.1519
	1.2	−0.7320

**Table 5:** Change in Sherwood number with the change in Brownian motion parameter, Lewis number and chemical reaction parameter.

$N_{b}$	Le	$\lambda_{CR}$	$Sh_{x}$
0.25			−0.5907
0.50			−2.9999
0.75			−3.5173
1.00			−3.6335
	0.2		3.9725
	0.4		3.1088
	0.6		2.3334
	0.8		1.6400
		1	−1.4594
		3	−2.9978
		5	−4.0786
		7	−4.9202

Table 6 is provided to examine the change in the motile microorganisms' density number against bioconvection parameters. This Table leads towards the fact that an increase in the microbes' parameter and Schmidt as well as Peclet number cause an enhancement in the motile microorganisms’ density number. Mass concentration of organisms is relatively greater than water. However, it raises the mass diffusivity and subsequently upsurges the diffusion rate of microorganisms.

**Table 6:** Change in motile microorganisms’ density number with the change in bioconvection Peclet number, Schmidt number and microorganisms parameter.

Pe	Sc	Ω	$Nn_{x}$
0.5			−3.1385
1.0			−3.9363
1.5			−4.7519
2.0			−5.5807
	2		−3.6584
	4		−6.4133
	6		−9.2369
	8		−12.1002
		2.0	−3.7689
		2.5	−3.9876
		3.0	−4.2063
		3.5	−4.4250

## 5 Conclusions

Interaction of nanoparticles with the gyrotactic microorganisms in a Darcy Forchheimer flow is examined numerically via the Successive over Relaxation technique. Some other prominent effects in the flow have also been taken into account. The insertion of microorganisms in the nanofluids improves the thermal properties of the fluid and causes a stability in the flow. We enlist the main results as follows:

➢ A significant enhancement is noticed in temperature in case of an increase in the values of heat generation and thermophoresis parameter.
➢ Both normal velocity F(ξ) and streamwise velocity $F^{\prime }\left(\xi \right)$ are decelerated by the Darcy Forchheimer parameter $D_{F}$.
➢ The concentration profiles seem to be diminishing with the effect of chemical reaction parameter and Lewis number.
➢ The bioconvection parameters such as Lewis number Le, motile microbes' parameter Ω and Peclet number Pe tend to depress the microbes' density G(ξ).
➢ An increase in the microbes' parameter and Schmidt as well as Peclet number cause an enhancement in the motile microorganisms' density number.
➢ The heat transport rate is reduced by the thermophoretic and heat generation parameter.

## Author contribution statement

Faiz Muhammad: Conceived and designed the experiments; Performed the experiments; Analyzed and interpreted the data; Contributed reagents, materials, analysis tools or data; Wrote the paper.

Tahar Tayebi: Analyzed and interpreted the data; Contributed reagents, materials, analysis tools or data; Wrote the paper.

Kashif Ali, Sohail Ahmad: Conceived and designed the experiments; Analyzed and interpreted the data; Contributed reagents, materials, analysis tools or data; Wrote the paper.

Emad Hasani Malekshah: Contributed reagents, materials, analysis tools or data; Wrote the paper.

## Data availability statement

Data will be made available on request.

## Declaration of competing interest

The authors declare that they have no known competing financial interests or personal relationships that could have appeared to influence the work reported in this paper.
